# Supplementary material for: Roles of S-Adenosylmethionine and Its Derivatives in Salt Tolerance of Cotton
Source: Int J Mol Sci. 2023 May 30;24(11):9517. doi: 10.3390/ijms24119517 (PMC10253415; doi:10.3390/ijms24119517)
Supplement: Supplementary file 1 [file ijms-24-09517-s001.zip › Materials and methods in the dissection of the molecular function of GhSAMC.pdf]

Materials and methods

Plant materials, growth conditions and treatment methods

G. hirsutum Texas Marker-1 (TM-1) was cultivated in the greenhouse with 14 h light /10 h dark and 50% relative humidity at 25◦C. When the third true leaf was unfolded (about 4 weeks), it was treated with NaCl (250 mM) solution. Samples were taken at 0, 1, 3, 6, 12 and 24 h, respectively. All samples were immediately placed in liquid nitrogen and stored in -80℃ refrigerator.

RNA extraction and fluorescence quantitative PCR

The cotton total RNA was extracted using RNAprep Pure Plant Plus Kit (TIANGEN, Beijing, China). First-strand synthesis of cDNA was performed using *TransScript*® Uni All-in-One First-Strand cDNA Synthesis SuperMix for qPCR (TransGen, Beijing, China). *PerfectStart*® Green qPCR SuperMix (TransGen, Beijing, China) and QuantStudio 3 Real-Time PCR System (Applied Biosystems) were used to carry out quantificational real-time polymerase chain reaction (qRT-PCR) experiments. The protocol was performed as follows: step 1: 94◦C for 30 s; step 2: 40 cycles of 94◦C for 5 s, and 60◦C for 30 s; and step 3: melting curve analysis. *GhHis3* (AF024716) was used as an internal reference gene[1] .Three biological repeats for each sample were performed , and the relative gene expression values were calculated using the $2^{-\triangle\triangle CT}$ method[2]. The primers used in qRT-PCR are shown in Supplementary Table S1.

Virus-induced gene silencing

The *GhSAMC* (*Gh_A05G2087*) fragments of 350 nt was cloned into the pTRV2 vector. The recombinant vector was transformed into Agrobacterium tumefaciens GV3101. *Agrobacterium* cells were resuspended in infiltration media (10 mM $MgCl_2$, 10 mM MES, 200 μM acetosyringone) and adjusted to OD_{600} = 1.0. We injected GV3101 bacterial solution carrying pTRV2 (empty vector), pTRV2-GhSAMC, pTRV2-CLA1 (positive control) and pTRV1 (helper vector) into the cotyledons of TM-1 according to the method mentioned by Tuttle[3]. After 24 h of dark treatment, the cotton plants were moved to a greenhouse (25◦C, 14 h light/10 h dark, 50% relative humidity). qRT-PCR was performed to further confirm that *GhSAMC* had been silenced in VIGS experiments. Then, these *GhSAMC*-silenced plants and control plants (TRV:00) were treated with 250 mM NaCl solution until the phenotypes appeared. The primers used are listed in Supplementary Table S1.

Measurement of MDA and T-AOC content

To detect MDA and T-AOC content , 0.1g leaves were collected from the GhSAMC-silenced and control plants (TRV:00) (treated with 250 mM NaCl solution) respectively, three biological repeats for each sample. The leaves were ground to homogenate on ice. For quantification of MDA and T-AOC contents, leaves of cotton were prepared and followed the procedure as described in Micro Malondialdehyde (MDA) Assay Kit (Solarbio, Beijing, China)and Total Antioxidant Capacity (T-AOC) Assay Kit (Solarbio, Beijing, China) , respectively.

Transcriptome analysis

The leaves of VIGS plants (TRV:SAMC, TRV:00) treated with 250 mM of NaCl solution were collected for transcriptome sequencing after one week salt treatment, three biological repeats for each sample. Shanghai Personalbio Technology Co., Ltd was entrusted to complete RNA extraction, library construction and transcriptome sequencing. FastQC software was used to analyze the quality of the original data, and then cutadapt was used to remove the low-quality bases to obtain Clean Reads. The clean RNA-seq reads were mapped to the G.hirsutum TM1-CRl v1.0 Genome using HISAT2 to obtain SAM format data[4]. Using SAMtools to convert SAM format data into BAM format data. The obtained transcripts were quantitatively analyzed using subread. The differentially expressed genes were analyzed by DESeq2 package of R language, and $P \leq 0.05$, $| \log2$ foldchange $| \geq 1$ was the condition for screening differentially expressed genes[5]. R language was used to calculate the Fragments Per Kilobase of exon model per Million mapped fragments(FPKM) value and draw the heat map. TBtools was used to perform GO and KEGG enrichment analysis on the differential expressed genes[6].

Data availability

The sequencing data for this study have been deposited into the Sequence Read Archive under accession PRJNA933089 and PRJNA937453.

References:

1. Liu, Z.; Ge, X.; Yang, Z.; Zhang, C.; Zhao, G.; Chen, E.; Liu, J.; Zhang, X.; Li, F. Genome-wide identification and characteriza tion of SnRK2 gene family in cotton (*Gossypium hirsutum* L.). BMC Genet. **2017**,18(1):54. doi:10.1186/s12863-017-0517-3.
2. Livak, K.J.; Schmittgen, T.D. Analysis of Relative Gene Expression Data Using Real-Time Quantitative PCR and the $2^{-\Delta\Delta CT}$ Method. *Methods.* **2001**, 25, 402-408, doi:10.1006/meth.2001.1262.
3. Tuttle, J.R.; Haigler, C.H.; Robertson, D.N. Virus-induced gene silencing of fiber-related genes in cotton. *Methods Mol Biol*.**2015**, 219-234, doi:10.1007/978-1-4939-2453-016.
4. Pertea, M.; Kim, D.; Pertea, G.M.; Leek, J.T.; Salzberg, S.L. Transcript-level expression analysis of RNA-seq experiments with HISAT, StringTie and Ballgown. *Nat. Protoc.* **2016**, 11, 1650-1667, doi:10.1038/nprot.2016.095.
5. Anders, S.; Pyl, P.T.; Huber, W. HTSeq-a Python framework to work with high-throughput sequencing data. *Bioinformatics* **2015**, 31, 166-169, doi:10.1093/bioinformatics/btu638.
6. Chen, C.; Chen, H.; Zhang, Y.; Thomas, H.R.; Frank, M.H.; He, Y.; Xia, R. TBtools: An Integrative Toolkit Developed for Interactive Analyses of Big Biological Data. *Mol. Plant.* **2020**, 13, 1194-1202, doi:10.1016/j.molp.2020.06.009.
