# Supplementary figures and images for: Roles of S-Adenosylmethionine and Its Derivatives in Salt Tolerance of Cotton
Source: Int J Mol Sci. 2023 May 30;24(11):9517. doi: 10.3390/ijms24119517 (PMC10253415; doi:10.3390/ijms24119517)

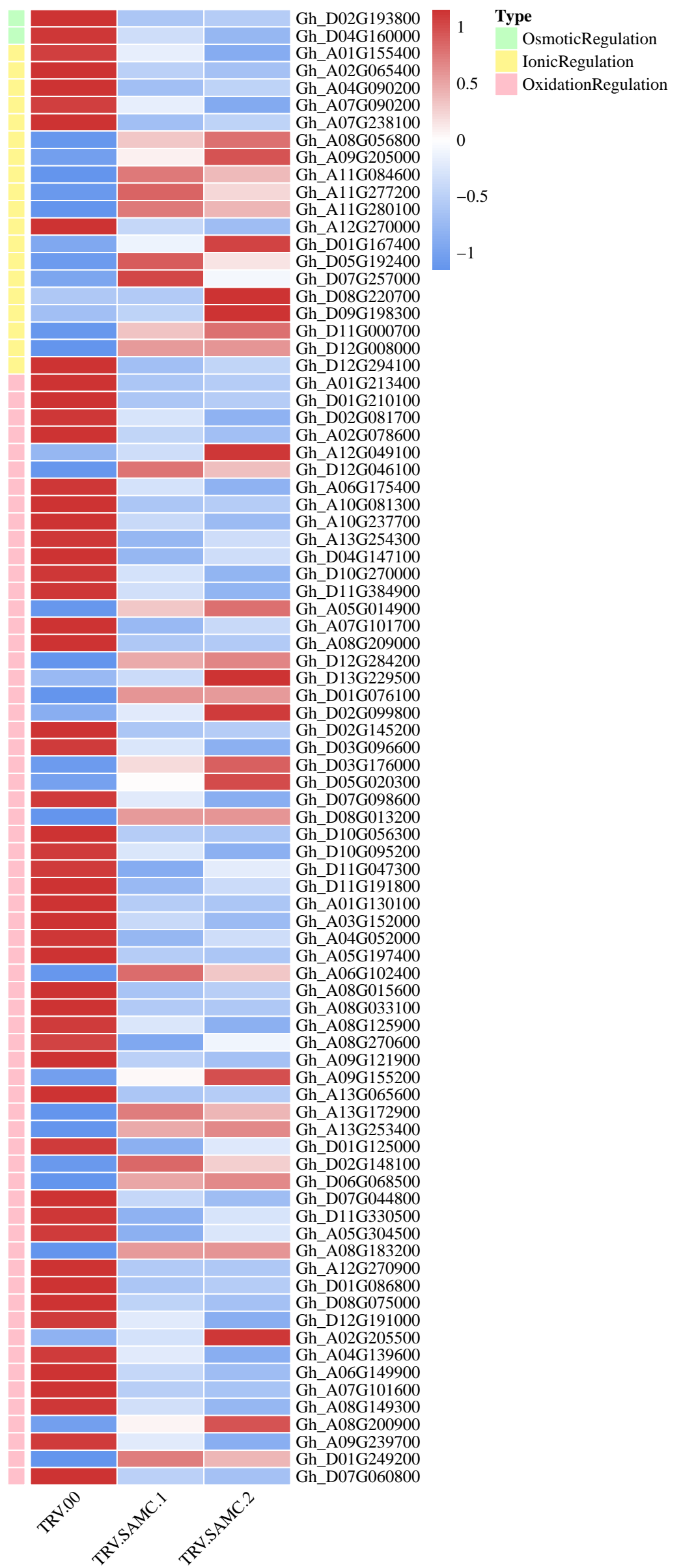

Supplement: Supplementary file 1 [file ijms-24-09517-s001.zip › Supplementary Figure S2. Differentially expressed genes related to osmotic regulation, ion regulation and oxidative regulation.pdf]
