# Supplementary material for: Roles of S-Adenosylmethionine and Its Derivatives in Salt Tolerance of Cotton
Source: Int J Mol Sci. 2023 May 30;24(11):9517. doi: 10.3390/ijms24119517 (PMC10253415; doi:10.3390/ijms24119517)
Supplement: Supplementary file 1 [file supplementary Figure S1. Protein sequence alignment for GhSAMC, AtSAMC1 and AtSAMC2.pdf › supplementary Figure S1. Protein sequence alignment for GhSAMC, AtSAMC1 and AtSAMC2.pdf]

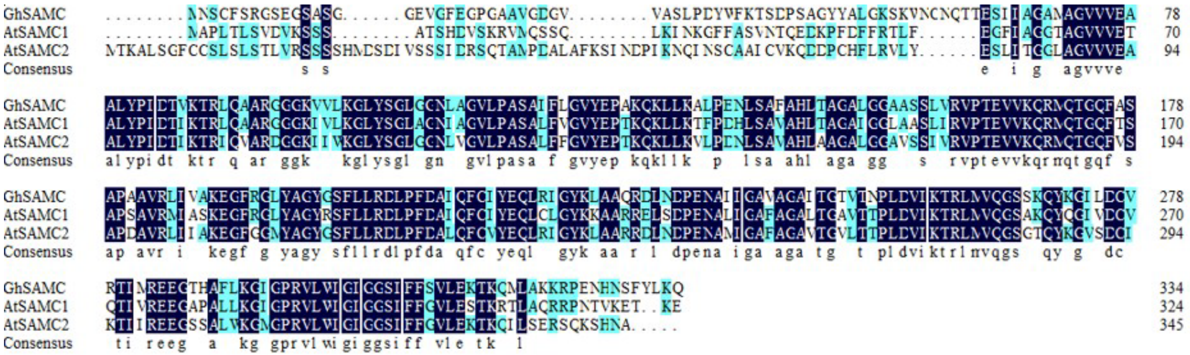
GhSAMC
AtSAMC1
AtSAMC2
Consensus
78
70
94
178
170
194
278
270
294
334
324
345
